# Electrochemical Preparation of Synergistic Nanoantimicrobials

**DOI:** 10.3390/molecules25010049

**Published:** 2019-12-22

**Authors:** Maria Chiara Sportelli, Daniela Longano, Elisabetta Bonerba, Giuseppina Tantillo, Luisa Torsi, Luigia Sabbatini, Nicola Cioffi, Nicoletta Ditaranto

**Affiliations:** 1 Dipartimento di Chimica, Università degli Studi di Bari Aldo Moro, via Orabona 4, I–70125 Bari, Italy; maria.sportelli@uniba.it (M.C.S.); daniela.longano@uniba.it (D.L.); luisa.torsi@uniba.it (L.T.); luigia.sabbatini@uniba.it (L.S.); 2 Dipartimento di Fisica, Istituto di Fotonica e Nanotecnologie UOS Bari, CNR, Via Amendola 173, I–70126 Bari, Italy; 3 Dipartimento di Medicina Veterinaria, Università degli Studi di Bari Aldo Moro, Strada Prov. 62 per Casamassima, Km 3, I–70010 Valenzano (BA), Italy; elisabetta.bonerba@uniba.it (E.B.); giuseppina.tantillo@uniba.it (G.T.)

**Keywords:** copper nanoparticle, benzalkonium chloride, synergistic antimicrobial, nanomaterial, XPS, TEM, ETAAS

## Abstract

The rapid spreading of resistance among common bacterial pathogens towards the misused antibiotics/disinfectant agents has drawn much attention worldwide to bacterial infections. In light of this, the present work aimed at the realization of core–shell nanoparticles possessing remarkable antimicrobial properties thanks to the synergistic action of the metal core and the disinfectant shell. Copper nanoparticles stabilized by benzalkonium chloride were prepared, characterized, and implemented in poly-vinyl-methyl ketone to obtain nanoantimicrobial composite coatings. Bioactivity tests are reported, proving the excellent disinfectant properties of the proposed nanomaterials, as compared to one of the well-known and strongest silver-based nanoantimicrobials. Applications are also briefly described.

## 1. Introduction

Nowadays nanomaterials are considered as an innovation in all technological and industrial fields, from the medical, biomedical, and pharmaceutical sectors to the electronic and agri-food areas [1]. Besides this, nanomaterials have revolutionized the world of antimicrobial agents. In fact, after the introduction of penicillin, molecular antibiotics became the standard treatment for bacterial infections [2,3]. Regrettably, the misuse of molecular antibiotics rapidly led to the evolving of the “antimicrobial resistance” (AMR) phenomenon [4], as highlighted by the World Health Organization (WHO) [5]. Since nanoparticles’ mode of action is the result of simultaneous processes (production of reactive oxygen species (ROS), electrostatic interaction with the cell membrane, internalization, ion release, etc.), most of the resistance mechanisms occurring with classic antibiotics are ineffective in the case of nanoparticles (NPs) [3,6]. Therefore, the new strategies against multidrug resistant microorganisms are more and more based on selected antimicrobials, such as antibiotics and/or peptides. In this regards, multimetal NPs can offer a suitable alternative [7,8,9,10,11,12,13,14,15,16].

The production of highly pure metal nanocolloids becomes extremely important when they are needed as antimicrobials. In fact, NPs must be free from toxic chemicals and have controlled dimensions in order to avoid (nano)toxicological issues for humans [17,18]. Among all synthetic routes to metal nanoparticles, sacrificial anode electrolysis (SAE) combines the advantages of physical routes with the ease of experimental protocols typical of classical chemical approaches [19]. In this paper, we focused on the electrochemical synthesis of copper nanoparticles (CuNPs) stabilized by benzalkonium chloride (BAC). The latter belongs to the quaternary ammonium compounds, also known as ′quats′, having a broad spectrum of antimicrobial activity and used as disinfectants in hospitals [20]. BAC in particular is an asymmetric alkyl ammonium salt, well-known for its antimicrobial and antibiofilm properties [21].

Electrochemically synthesizing CuNPs in the presence of BAC, we aimed at obtaining CuNPs@BAC with enhanced and synergistic antimicrobial properties. Back to 2008, Harrison et al. demonstrated that Cu^2+^ ions can work synergistically with quaternary ammonium compounds to prevent biofilm adhesion and proliferation, reducing the activity of nitrate reductases, which are important enzymes for normal biofilm growth [22]. Analogously, Jaramillo et al. and Houari et al. showed that BAC can limit bacterial adherence in biofilm formation, inhibiting the conditioning layer development [23,24].

In most of antimicrobial applications, the effectiveness of the intervention can be reduced by the partial inactivation of the biocidal compound overtime. Therefore, the use of synergistic antimicrobials might overcome these limitations and improve efficacy [25]. In the specific case of our CuNPs@BAC, they can be considered as core–shell nanostructures, i.e., having a core made of Cu and a BAC shell, which encloses the aforementioned core. Hence, the core is a metal nanophase having intrinsic antimicrobial properties, and the shell is made of a quaternary ammonium compound, preferably a surfactant agent, with biocide activity. Under these circumstances, BAC can act both as NP stabilizer (preventing aggregation and improving NP dispersibility in various matrices), and as disinfectant [26].

Following our previous work about the SAE preparation of CuNPs [19,27,28,29], here we proposed the electrochemical preparation of CuNPs stabilized by benzalkonium chloride and their implementation in polyvinyl-methyl-ketone (PVMK) thin films. NPs were characterized by transmission electron microscopy. NPs and thin films were analyzed by X-ray photoelectron spectroscopy in order to achieve surface chemical composition and chemical speciation. A systematic evaluation of copper release properties in aqueous solutions was performed as a function of the CuNPs loading with atomic absorption spectroscopy. Finally, CuNPs@BAC antimicrobial activity was tested against model microorganisms, i.e., *Staphylococcus aureus* and *Escherichia coli*, in comparison with a well-known silver-based disinfectant agent.

## 2. Results and Discussion

### 2.1. Nanomaterials Preparation and Morphological Characterizaztion

The electrochemical preparation of CuNPs stabilized by benzalkonium chloride (CuNPs@BAC) was achieved by sacrificial anode electrolysis (SAE) [19,26,27]. The set-up for electrochemical synthesis and the mechanism of CuNPs formation are reported elsewhere [19]. Briefly, the potential applied to the working electrode let the anode be oxidized. Anode corrosion results in the production of cupric ions that are then reduced at the cathode surface, where they are subsequently stabilized by the surfactant. The result is the formation of core–shell NPs dispersed in the solution. In this paper, BAC was used as both electrolyte and capping agent. BAC is a cationic surfactant belonging to the quaternary ammonium salts and usually is a mixture of alkyl-benzyl-dimethyl ammonium chlorides, in which the length of the alkyl chain can vary by up to 18 carbon atoms. In this study, hexadecyl-benzyl-dimethyl ammonium chloride was used as CuNPs stabilizer. With respect to previous alkyl ammonium salts already used as CuNPs stabilizers [19,30], BAC has only one long alkyl chain, therefore it is expected to form a less uniform shell around the copper core, resulting in a worse stabilizing character. Indeed, SAE of this nanomaterial required an optimization of the electrochemical parameters in terms of surfactant concentration (from 0.01 to 0.2 M) and working potential (from 0.5 to 2.5 V). The morphology of the colloidal dispersions was investigated by TEM studies and the results are reported in Table S1.

**Figure 1:**
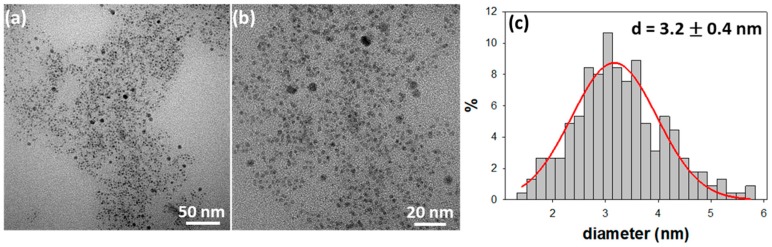
TEM images (**a**,**b**) and size distribution histogram (**c**) of the electrosynthesized copper nanoparticles (CuNPs) stabilized by benzalkonium chloride (BAC)(CuNPs@BAC) (0.1 M, 1.5 V). The mean NP core diameter is reported in the inset, along with the relevant standard deviation (n = 225).

Figure 1 reports the images and the size distribution histogram of CuNPs@BAC particles electrosynthesized with BAC 0.1 M and a working potential of 1.5 V.

TEM pictures revealed the presence of spheroidal nanoparticles having an average core diameter of 3.2 ± 0.4 nm. Unfortunately, these CuNPs did not show a very long-term morphological stability since some precipitation and aggregates formation was observed upon storage for a few weeks. Changing of the SAE parameters led to a slight improvement in the morphological stability of the nanocolloids in terms of aggregation and precipitation, but did not significantly affect the average size of the suspended colloidal fraction (see Table S1). Moreover, the use of BAC concentrations higher than 0.1 M may affect the nanocolloid processing when thin coatings have to be obtained, because of the excessive presence of hygroscopic quaternary ammonium species in the NP suspension. Therefore, the experimental parameters used to prepare the dispersion of Figure 1 represent an acceptable compromise between processability and stability towards storage. Indeed, when the BAC concentration was increased, the greater relative abundance of the organic/stabilizing component in the colloid could help to preserve the copper core at zero oxidation state, at least in the as-prepared CuNPs. On the other hand, this turned out to be irrelevant in any real life application step, as the subsequent air exposure of nanoparticles—as-prepared or dispersed in polymer matrices —lead them to a rapid oxidation to CuO, which still represents a key intermediate in the mechanism of corrosion and copper ionic release [19,27].

Dynamic Light Scattering (DLS) and ζ potential measurements were also performed on the colloidal dispersions. Results showed DLS diameter to be strongly dependent on the lifetime of the NPs. The value typically ranged from a few nanometers for freshly prepared colloids (comparable with the size diameter found in TEM measurements) up to 1.5 μm after 3–4 months of ageing. The ζ potential value was generally a positive value in the range 60–90 mV, reflecting the nature of the cationic surfactant shell. The specific value for CuNPs@BAC of Figure 1 is 80 ± 4 mV.

Nevertheless, the interesting antiseptic properties of BAC and the envisaged synergistic antimicrobial effects in combination with CuNPs led to a compromise on their limited stability. Proper amounts of CuNPs@BAC were therefore mixed into the PVMK solutions to obtain mixed solutions for depositing nanostructured composite films at different copper/polymer weight percentages, in the range from 0.5 to 10.0%*_w/w_*.

### 2.2. Spectroscopic Characterizaztion

CuNP dispersions and Cu-based PVMK composites were analyzed by XPS in terms of atomic percentages (El%) and surface chemical speciation. In order to investigate the colloids’ chemical stability, XPS analysis was performed on fresh CuNP samples and on 29-day-old ones. The El% of both CuNPs@BAC are reported in Table 1.

The elements revealed on the surface were expected based on the chemical composition of the CuNPs: copper, along with carbon, nitrogen, and chlorine from the surfactant. Small amounts of oxygen were also detected on as-prepared samples, possibly due to surface organic contamination. As a matter of fact, the ageing period led to a marked increase of %O caused by the copper oxidation; Cu% also increased, mainly due to a surfacing process. Both findings confirmed the low stabilizing property of BAC over a long period.

**Figure 2:**
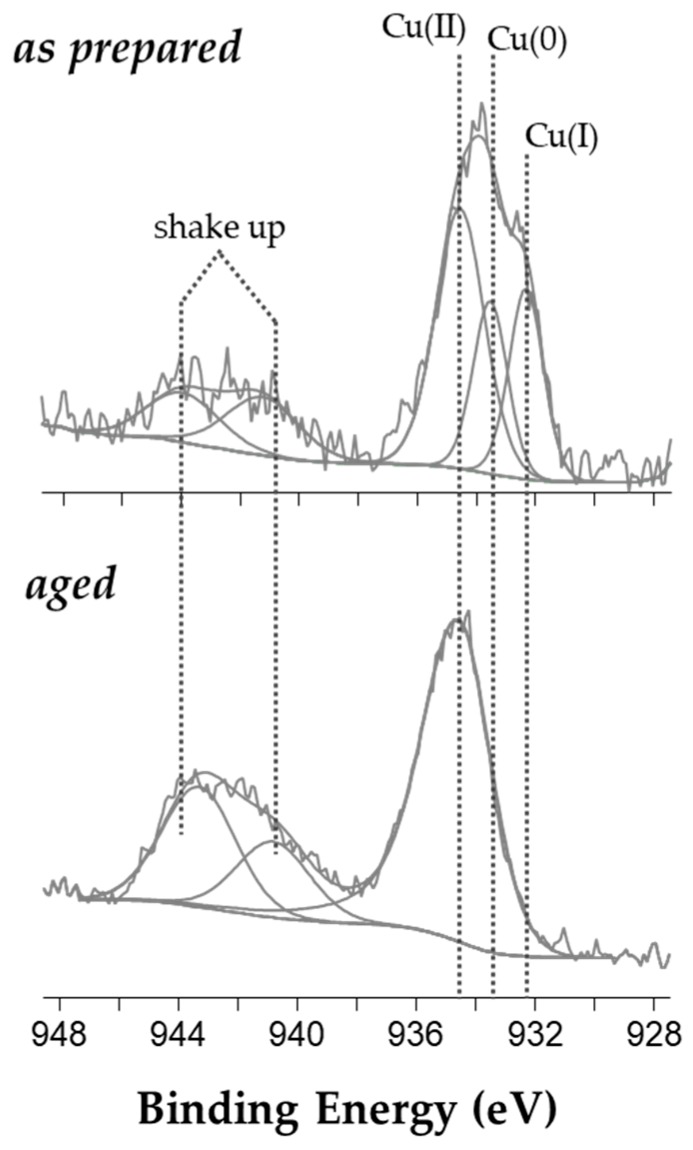
Cu2p_3/2_ XP high resolution spectra of as-prepared and 29-day-old CuNPs@BAC.

Copper chemical speciation was then studied after a curve-fitting procedure of XP Cu2p_3/2_ spectral region of both as-prepared and aged colloid (Figure 2).

In particular, the signal analysis of the spectrum relevant to a pristine nanocolloid resulted in five peak components: two shake-up features (spectral region, 940–945 eV) and a photoelectron peak at 934.5 ± 0.2 eV, recalling the presence of Cu^2+^ species [31]. Two more components were found at 933.5 ± 0.2 and at 932.3 ± 0.2 eV, and respectively ascribed to nanodispersed Cu(0) [19,32,33] and to CuCl^+^ complexes (also evident from XP Cl2p region, data not shown). After ageing in air, the Cu2p_3/2_ signal shape appeared to be different and the curve fitting resulted in two shake-up shoulders and only one photoelectron peak at 934.5 ± 0.2 eV. All those components were attributed to Cu(II), revealing the complete oxidation of copper cores in less than one month of air exposure.

The as-prepared CuNPs colloid was used for the preparation PVMK-based composite films with different Cu%*_w/w_*, namely 0.5%, 1.0%, 2.0%, 5.0%, and 10.0%. Their surface chemical composition is summarized in Table 2. The composite film with an intermediate copper loading (2.0%) was also investigated after one month of ageing in air.

From the comparison of the El% data shown in Table 2 it can be observed that %Cu increased with the nanoparticles loading. Also, an increase in the percentages of carbon, nitrogen, and chlorine, and a considerable decrease in the percentage of oxygen, were registered. The correlation between the surface copper availability and the amount embedded in the bulk of the composite is better highlighted by the copper to carbonyl ratio in the last column of Table 2, considering %C=O as a target component of the polymer. The increase of %Cl and %N, affected by the contribution of the stabilizing surfactant of CuNPs, was observed as well. A comparison of surface percentages relevant to as-prepared and aged nanocomposite coatings does not show any significant change in the copper surface availability, and this is reasonably due to the stabilizing effect of the polymer dispersing the bioactive nanophases.

Previous studies on nanomaterials stabilized by quaternary alkyl ammonium salts demonstrated a controlled copper release when they are put in contact with aqueous solutions, thus exerting a marked inhibiting action against the growth of various copper-sensitive microorganisms [2,19,27,34,35,36]. In this work, a systematic evaluation of copper release properties in aqueous solutions of composite films stabilized by BAC disinfectant agent was performed as a function of the CuNPs loading. Table 3 reports the atomic absorption analyses (ETAAS) experimental results.

A general modulation of the release amount as a function of the nanoparticle loading in the composite material could be observed. Only for the nanomaterial loaded with 5.0% of CuNPs, the plateau value was lower than expected, although the discrepancy with respect to the general trend can be interpreted in terms of the high sample-to-sample variability (highest standard deviation). All the release trends were interpolated according to a kinetic model of the pseudo-first order (data not shown). The relevant kinetic constants, listed in the third column of Table 3, were not significantly different, demonstrating that the release kinetics were not affected by the NPs loading.

Release experiments, carried out on aged composites, showed similar results, in terms of trend release and modulation.

The copper concentration plateau values obtained for these nanomaterials are in line with those obtained in previous studies [19,27], therefore an antimicrobial action is expected from the nanoantimicrobials developed in this work.

### 2.3. Biological Results

Several protocols are available to test the antimicrobial activity of a specific agent, each of them developed and used according to the requirements of the single experiment to be carried out. In previous works, we proved the biostatic/bioactive efficacy of CuNPs by embedding them in dispersing matrices and evaluating the proliferation of the microorganisms in correlation with the ionic copper release from the coatings [19,27]. In this study, a double approach was followed.

In a first experiment, PVMK-based nanocomposite coatings embedding different copper materials/alkyl ammonium salts were put in contact with a bacterial broth. In order to discriminate a possible synergistic action of CuNPs in combination with BAC, nanocomposites containing 5.0%*_w/w_* of (a) Cu^2+^ salt (CuCl_2_), (b) tetra-butyl-ammonium perchlorate stabilized CuNPs (CuNPs@TBAP), (c) CuNPs@BAC, and (d) 35.0%*_w/w_* of BAC were tested. The results reported in Table 4 showed that the proliferation of the copper resistant microorganism strain (*E. Coli ATCC 25922*) was slowed down when a huge amount of BAC was dispersed in the coating and was completely inhibited thanks to the combination of CuNPs and BAC. The presence of copper ions released from a copper salt or from a different core–shell NP was not able to affect the growth of this copper-resistant bacterial strain, while CuNPs@BAC exerted a strong synergistic antibacterial action.

In a second experiment, the biological results were obtained by evaluating the minimum inhibitory concentration (MIC) of the antimicrobial agent needed to completely inhibit microorganisms’ growth and proliferation. The antimicrobial activity tests were performed comparing the performance of CuNPs@BAC samples with those of a model synergistic nanoantimicrobial, namely, myramistin-capped silver nanoparticles, AgNPs@Myr, which has been reported to be a strong disinfectant nanomaterial [37].

MIC values were expressed as the amount of the antimicrobial agent (μg/mL) able to inhibit the microorganism growth. The MICs were then evaluated using a fixed amount of bacterial suspension supplemented with different concentrations of tested nanomaterials. The MICs of the tested samples against the chosen bacterial resistant strains are summarized in Table 5.

As shown in Table 5, at least 25 μg/mL of AgNPs@Myr were requested to completely inhibit the growth of microorganism #1, compared to 12.5 μg/mL of CuNPs@BAC. Similarly, less than 1 μg/mL of CuNPs was enough against microorganism #2 proliferation, instead of 3.125 μg/mL of AgNPs. Finally, microorganism #3 was inhibited by only 3.125 μg/mL of CuNPs, compared to 25 μg/mL of AgNPs@Myr.

Therefore, CuNPs@BAC proved to be more effective than AgNPs@Myr when used as antimicrobial agent against the tested bacterial strains, since in all the cases more diluted CuNPs dispersions were used to completely inhibit microorganisms growth and proliferation.

**Figure 3:**
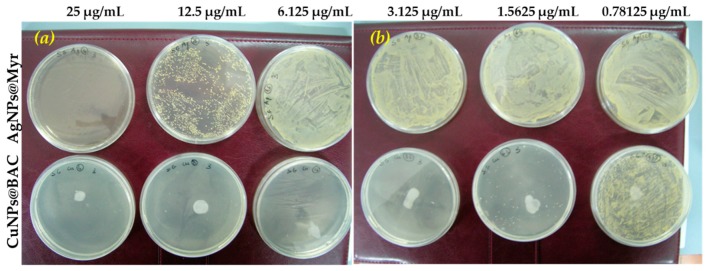
Petri plates for microorganism #3 incubated with AgNPs (upper line in (**a**,**b**)) and CuNPs samples (bottom line in (**a**,**b**)). (Please note that the big white circle in each plate supplemented with CuNPs is not a bacterial colony, but is due to the plastic of Petri plate tarnished by the contact with the nanomaterial).

Visual results are presented in Figure 3.

Figure 3a,b show the Petri plates obtained by uniformly distributing 50 μL 10^7^ UFC ml^−1^ suspension of #3 bacterial strain on the solid G medium surface supplemented with tested AgNPs or CuNPs after 24 h incubation at 37 °C. As can be observed, high dilutions of AgNPs nanomaterial (16–128 times, see Table S2 for correspondance) let a very high number of colonies to grow up: no dots can be distinguished but a yellow continuous patina formed on the surface of the plates (pictures on the upper line of Figure 3a,b). A slight antimicrobial effect was performed with the nanomaterial diluted 8 times, while the complete inhibition is performed at higher AgNPs concentration, that is MIC = 25 μg/mL.

On the contrary, CuNPs@BAC nanomaterial was already effective at very low concentrations (1.5625 μg/mL), causing the complete plate disinfection at MIC = 3.125 μg/mL.

Based on the remarkable antimicrobial action proved by CuNPs@BAC, they were used for the modification of real life products for a proof of concept experiment. Specifically, three textile products having different cotton/synthetic fiber composition, were modified with the nanocolloid at two different CuNPs concentrations and were used in copper release experiments. In all the cases, a significant Cu plateau concentration value was registered. Also, for each kind of fabric, the higher the CuNPs concentration in the impregnation bath the higher the copper release, in accordance with the typical behavior of similar core–shell nanoparticles [19] (Table S3).

## 3. Materials and Methods

### 3.1. Preparation of CuNPs@BAC and CuNPs-PVMK

The synthesis was performed in a conventional three electrode cell: the working electrode consisted of a copper plate and the counter electrode was a platinum plate, both with thickness of 0.5 mm and area of 2.5 cm^2^ for an overall solution volume of 10 mL. Reference electrode was a lab made Ag/AgNO_3_ 0.1 M in acetonitrile. The electrosynthesis solution contained benzalkonium chloride (BAC) dissolved in a mixture of acenonitrile (ACN) and tetrahydrofuran (THF) in proportion 1:3. BAC is commercially available (CAS number 63449-41-2). The electrosynthesis was carried out at room temperature and under nitrogen atmosphere to prevent NP oxidation. The typical electrolysis time was 3 h, with an average current of 7 mA.

Copper nanocomposites were prepared as follows: CuNPs colloidal dispersion was mixed at a fixed ratio to a solution of poly-vinyl-methyl ketone (PVMK, Aldrich, Mw 500,000) in ACN:THF 1:3 at a concentration of 25 g L^−1^. The resulting solutions were directly spin-coated on a proper substrate for characterization and biological testing. The spin coating comprised a spreading phase at 350 rpm for 9 s and a subsequent drying phase at 2,000 rpm for 20 s.

### 3.2. Morphological Characterization

The TEM analyses of the CuNPs produced in this work were conducted using a FEI Tecnai Spirit G2 microscope (FEI Company, Hillsboro, OR, USA), equipped with a LaB6 electronic filament gun and operating at a voltage of 120 kV. Samples were diluted 1:100 before placing a drop on a copper grid (TAAB, carbon coated 300 mesh).

### 3.3. Spectroscopic Characterization

Metal slides deposited with films of CuNPs@BAC and CuNPs-PVMK nanocomposites were characterized by means of x-ray photoelectron spectroscopy (XPS), using a Theta Probe VG Scientific spectrometer (Thermo Fisher Scientific, East Grinstead, UK) equipped with a monochromatized AlKα source (spot = 400 μm). Survey spectra were recorded in constant analyzer energy mode (CAE) at a pass energy of 150 eV, while high-resolution regions (C1s, O1s, N1s, Cl2p, Cu2p_3/2_) were acquired in CAE mode at a pass energy of 100 eV. Calibration of the binding energy (BE) scale was performed by fixing the aliphatic component of the C1s signal at BE values of 284.8 ± 0.1 eV. Data processing was performed using Avantage software v. 5.967 (Thermo Fisher Scientific, East Grinstead, UK). Other data processing details and calculation of surface elemental percentages were performed as described in [30]

The determination of the amount of copper released from the nanocomposites into an aqueous contact solution was carried out through atomic absorption analyses (ETAAS). Copper release experiments were performed putting the coatings in contact with 1 mL saline solution (pH 6.8 in phosphate buffer) for 24 h. The solutions were sampled several times and subjected to ETAAS analysis after proper dilution with HNO_3_ 0.2%. ETAAS was performed with a Perkin-Elmer460 double-beam spectrophotometer (PerkinElmer, Waltham, MA, USA) equipped with a copper hollow cathode lamp and a graphite furnace. The thermal treatment of the samples was programmed as follows: step 1, up to 110 °C in 30 s, hold time 30 s; step 2, up to 1000 °C in 30 s, hold time 30 s; step 3, up to 2100 °C immediately, hold time 10 s; step 4, up to 2400 °C in 2 s, hold time 2 s. A calibration curve was obtained by proper dilutions of copper standard for AAS; the measurement results carried out in triplicate are expressed as mean value ± 1S (one standard deviation).

### 3.4. Bactericidal Test Protocol

The nutrition media were prepared as described in the following, according to the experimental procedure reported in [37]. First of all liquid Gause (G) medium was prepared: 10 g glucose, 5 g peptone, 3 tryptone, and 5 g NaCl were dissolved in 1 L of distilled water. Then solid Gause (G) medium was obtained by adding 20 g of agar to the liquid G medium. After heating and sterilization by autoclaving at 121 °C for 15 min, AgNPs or the CuNPs dispersions were then added to the medium to have 2–128 times dilution of AgNPs and CuNPs in the media. In Table S2 the initial concentrations of copper and silver colloidal samples are reported, along with all the dilutions prepared and the corresponding concentrations of the nanomaterials.

## 4. Conclusions

In this study, the use of BAC as a CuNPs stabilizing agent was investigated to obtain electrochemically prepared synergistic core–shell antimicrobial nanomaterials. A proper investigation of the best electrochemical parameters was carried out, proving that the relative quantities of the two antibacterial components of the synergistic nanoantimicrobial (copper and BAC) can be independently varied, without a significant alteration of both the properties and the dimensions of the copper nanophases.

TEM and XPS characterizations provided morphological and chemical information on the nanoantimicrobials prepared, while ETAAS analyses proved the ability of CuNPs@BAC/PVMK nanocomposites to release copper ions as a function of the copper loading.

The bioactivity tests proved both the synergistic action of CuNPs and BAC when combined in a core–shell structure and also the remarkable overall biostatic/biocidal activity as compared to well-known silver-based disinfectant system. Besides this, the SAE procedure results proved the possibility of modulating the bioactivity of the nanocolloid by simply varying the BAC concentration with the same amount of dispersed bioactive metal, without affecting the final CuNPs properties.

## Figures and Tables

**Table 1:** XPS surface elemental composition of CuNPs@BAC samples as prepared and aged for 29 days. The error in the atomic percentages is 0.2% for copper, 0.5% for all the other elements.

	%Cu	%C	%O	%N	%Cl
as-prepared	1.5	88.8	1.8	4.8	3.1
aged	7.1	69.4	16.2	5.1	2.2

**Table 2:** XPS surface elemental composition of the nanocomposite films at different copper loadings. The error in the atomic percentages is 0.2% for copper, 0.5% for all the other elements.

CuNPs-PVMK Loading %*_w/w_*	%Cu	%C	%O	%N	%Cl	Cu/C=O
0.5%	0.4	79.4	19.4	0.4	0.4	0.05
1.0%	0.9	82.4	14.5	1.1	1.1	0.09
2.0% *as-prepared*	1.4	85.2	10.2	1.3	1.9	0.51
2.0% *aged*	1.1	78.2	18.3	1.1	1.3	0.13
5.0%	1.2	83.0	9.4	2.2	4.2	2.0
10.0%	1.5	86.7	4.8	2.6	4.4	2.0

**Table 3:** *Plateau* values and kinetic constants for copper release from CuNPs-PVMK composite films prepared at different copper loading. Data obtained after interpolation according to a first order kinetic trend. The values were averaged out of three replicates ± 1S (one standard deviation).

CuNPs-PVMK Loading *_w/w_*_%_	[Cu]_plateau_/ppb	k/h^-1^
0.5%	40 ± 2	9 ± 1
1.0%	210 ± 20	10 ± 2
2.0%	430 ± 40	8 ± 5
5.0%	230 ± 90	10 ± 6
10.0%	535 ± 5	7 ± 3

**Table 4:** Number of colony forming units (CFU) of *E. Coli ATCC 25922* grown on different composite coatings.

Sample	CFU
Control (Petri dish without any coating)	uncountable
PVMK + CuCl_2_ 5.0%*_w/w_*	uncountable
PVMK + CuNPs@TBAP 5.0%*_w/w_*	uncountable
PVMK + BAC 35.0%*_w/w_*	230
PVMK + CuNPs@BAC 5.0%*_w/w_*	0

**Table 5:** Minimum inhibitory concentrations (MICs) of tested myramistin-capped silver nanoparticles (AgNPs) and CuNPs samples against three microorganisms strains.

MIC (μg/mL)					
*#1 E. Coli ATCC 25922*		*#2 St. MRSA 33591*		*#3 St. MRSA 25923*	
**AgNPs@Myr**	**CuNPs@BAC**	**AgNPs@Myr**	**CuNPs@BAC**	**AgNPs@Myr**	**CuNPs@BAC**
25	12.5	3.125	<1	25	3.125

## Supplementary Materials

The following are available online, Table S1: CuNPs@BAC morphological results obtained varying the electrochemical parameters during the preparation process, Table S2: AgNPs and CuNPs concentrations in the liquid G medium. Initial concentration was 10^-4^ g/mL (= 100 µg/mL), Table S3: Plateau values for copper release from three types of fabrics modified by CuNPs@BAC nanoparticles at two different CuNPs concentrations.
