# Supplementary material for: Electrochemical Preparation of Synergistic Nanoantimicrobials
Source: Molecules. 2019 Dec 22;25(1):49. doi: 10.3390/molecules25010049 (PMC6983245; doi:10.3390/molecules25010049)
Supplement: Supplementary file 1 [file molecules-25-00049-s001.pdf]

Supplementary Material

Table S1. CuNPs@BAC morphological results obtained varying the electrochemical parameters during the preparation process.

TEM image	Dimensional histogram	Electrochemical parameters
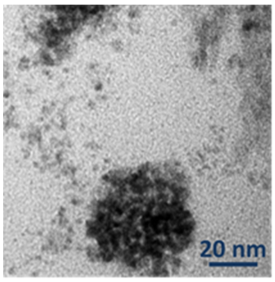	*-agglomerates-*	BAC=0.01 M<br>WP=1.5 V
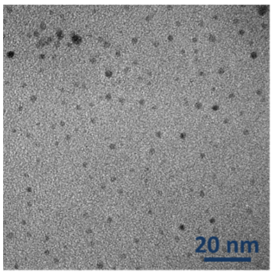	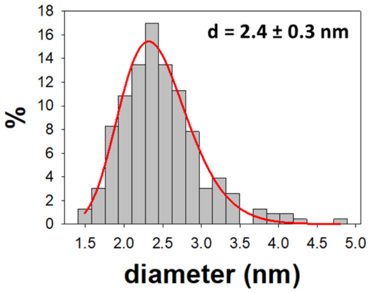	BAC=0.2 M<br>WP=1.5 V
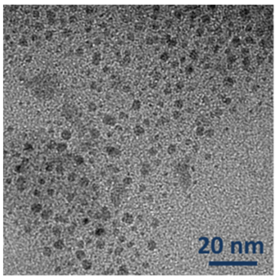	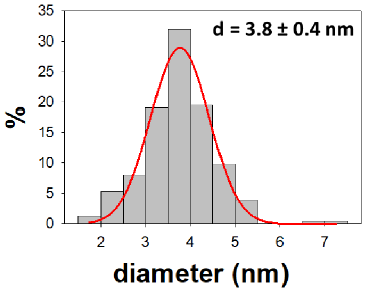	BAC=0.2 M<br>WP=2.5 V

Table S2. AgNPs and CuNPs concentrations in the liquid G medium. Initial concentration was 10^{-4} g/mL (= 100 μg/mL).

dilution	concentration / μg/mL
2	50
4	25
8	12.5
16	6.25
32	3.125
64	1.5625
128	0.78125

Table S3. *Plateau* values for copper release from three types of fabrics modified by CuNPs@BAC nanoparticles at two different CuNPs concentrations.

Fabric	CuNPs concentration / gL^{-1}	$[Cu]_{plateau}$ / ppb
(a) 100% polyestere	0.05	150±10
	0.5	160±10
(b) 60% cotton-40% polyestere	0.05	100±20
	0.5	290±20
(c) 35% cotton-65% polyestere	0.05	20±5
	0.5	60±5
